# Absence of the appendix discovered during childhood

**DOI:** 10.1186/2193-1801-3-522

**Published:** 2014-09-12

**Authors:** Michelle V Vincent, Alex Doyle, Sean Bernstein, Selma Jackman

**Affiliations:** Department of Surgery, Queen Elizabeth Hospital, Martindales Road, St Michael, BB1115 Barbados

## Abstract

Absence of the appendix is rare. Isolated cases are usually discovered in adult patients or cadavers. We report the case of a 14 year old boy who was found to have no appendix on laparotomy for assumed acute appendicitis and use this opportunity to highlight the growing surgical uses of this vestigial structure.

## Introduction

Absence of the vermiform appendix is not usually encountered during childhood. It is fortunate that this condition is rarely encountered given the growing surgical uses of the appendix.

## Case report

A 14 year old boy was referred to our surgical unit with a one day history of worsening right iliac fossa pain, associated with fever and three episodes of non-bilious vomiting. He was diagnosed in early infancy with renal tubular acidosis with associated normal renal function. On presentation he was noted to be mildly dysmorphic with associated microcephaly. He was apyrexial with a pulse rate of 87 beats per minute. Abdominal examination revealed right iliac fossa tenderness with guarding. Investigations included a urinalysis which showed a trace of blood and 2+ each for ketones and protein. A complete blood count demonstrated a white cell count of 5.4 × 10^3^.

**Figure 1:**
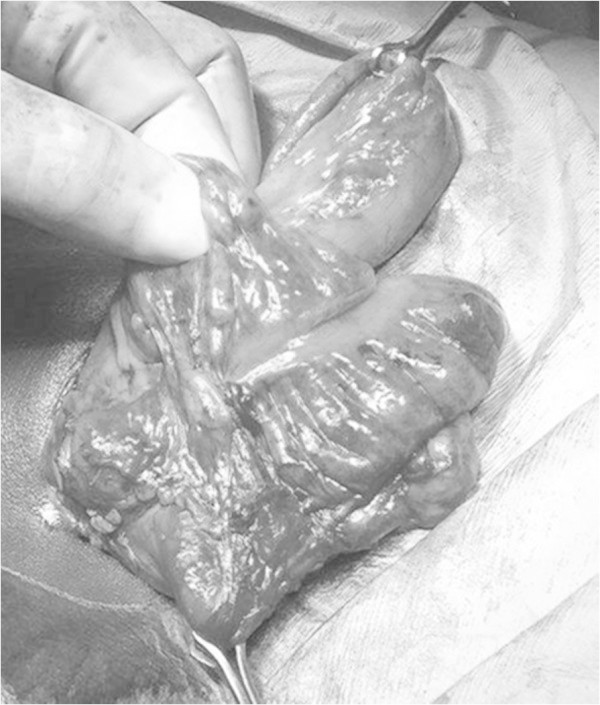
**Ileocecal junction fully mobilized with no apparent appendix.**

**Figure 2:**
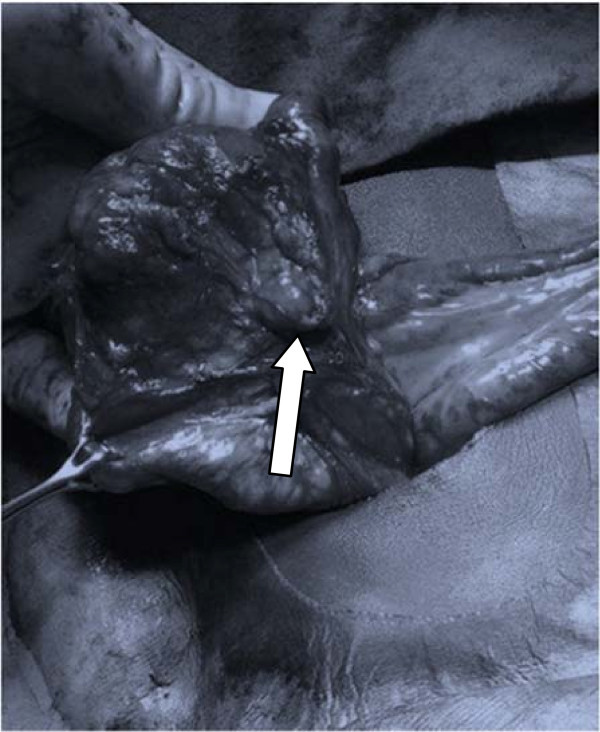
**Enlarged pericecal lymph nodes (white arrow).**

He was assessed as having an acute appendicitis and underwent laparotomy via a Lanz incision. Intraoperatively when the ileocecal junction was fully mobilized, including the retrocecal area, no appendix was apparent [Figure 1]. A careful search was also made for a meckel’s diverticulum, but none was present. Enlarged peri-cecal lymph nodes were noted [Figure 2]. The boy made a smooth postoperative recovery and was discharged home on the second postoperative day. On review two days later he was unexpectedly noted to be pyrexial with an associated wound infection, which was treated with regular wound irrigations and dressings until the wound was noted to be fully healed at two weeks follow-up.

## Discussion

Congenital absence of the appendix was first described by Morgagni in 1718 (Morgagni 1719; Greenberg et al. 2003) and is rarely encountered. The condition is found in 1 in 100,000 laparotomies for suspected acute appendicitis (Chevre et al. 2000; Sarkar 2012; Nissler et al. 2012; Lima et al. 2003; Host et al. 1972). Other congenital anomalies of the appendix include duplex appendix which has an incidence of 0.004%, and the even rarer finding of appendix triplex (Nissler et al. 2012). In addition there are few case reports of anomalous implantation of the appendix. For example, Scanavacca et al. in 2000 (Scanavacca et al. 2000) reported on the case of an 8 year old boy whose appendix was noted to arise from the anterior wall of the ascending colon, approximately 15 cm from the ileocecal valve.

The cause of an absent appendix is postulated to be secondary to an intrauterine vascular accident, (Hei 2003) as is noted in pediatric cases of intestinal atresias (Louw and Barnard 1955). This theory may be supported by the occasional findings of a fibrotic string-like structure within the peritoneal cavity in some cases where no appendix is found - referred to as autoamputation of the appendix, (Iuchtman 1993) and the even rarer phenomenon of appendiceal atresia (Woywodt et al. 1998; Yaylak et al. 2013). In addition, there are reports of jejuno-ileal atresias with associated absence of the appendix (Cserni 2006; Yokose 1986). The 1970’s also saw cases of appendiceal absence and atresia of the appendix associated with the use of Thalidomide- whose mechanism of action is postulated to be anti-angiogenic (Smithells 1978; Shand and Bremner 1977; Bremner and Mooney 1978).

At laparotomy or laparoscopy associated mesenteric lymphadenitis is sometimes noted (Chevre et al. 2000; Zetina-Mejia et al. 2009). However an autoamputated appendix may also be the focus for inflammation within the peritoneal cavity (Louw and Barnard 1955). In other cases no cause for the patient’s symptoms is found (Maitra et al. 2013; Rolff et al. 1992).

Most case reports of absence of the appendix are usually noted in adult patients (Greenberg et al. 2003; Chevre et al. 2000; Zetina-Mejia et al. 2009; Maitra et al. 2013; Rolff et al. 1992), or adult cadavers (Sarkar 2012; Host et al. 1972) but rarely in children (Nissler et al. 2012; Lima et al. 2003). The appendix itself is increasingly becoming an invaluable vestigial structure, particularly in pediatric surgical practice. At present it is used in the management of fecal (Malone et al. 1980) and urinary (Mitrofanoff 1980) incontinence, as well as for ureteral substitution (Martin 1981; Estevao-Costa 1999) and as a biliary conduit in the management of children with choledochal cysts and biliary trauma (Valla 1988; Sarin et al. 2007; Shah and Shah 2005).

When applied for use in the management of children with refractory constipation (with overflow incontinence) and fecal incontinence the appendix is mobilized, its distal end removed and the open end anastomosed to the skin of the anterior abdominal wall, typically at the umbilicus or in the right iliac fossa. Through this appendiceal channel washout enemas can be administered after which the child sits on the toilet to empty the bowel in a controlled manner. The enema is then repeated every day or on alternate days. During the intervals of enema administration the colon is empty and thus the child kept from being constipated or having episodes of fecal soiling/incontinence. Since its introduction in 1980 (Malone et al. 1980) this procedure- the Malone or MACE (Malone antegrade continence enema) has undergone many modifications and is now widely used in the management of refractory constipation and fecal incontinence in children with myelomeningoceles, neuropathic conditions for example spina bifida, anorectal malformations, Hirschsprung’s disease and chronic intractable constipation (Imai et al. 2014; Hoekstra et al. 2011; VanderBrink et al. 2013).

In the management of urinary incontinence access to a normal bladder, augmented bladder or continent reservoir can be obtained by creating a catheterizable channel between the bladder and skin using the appendix- the Mitrofanoff principle (Mitrofanoff 1980). Like the Malone procedure, the Mitrofanoff procedure has also undergone many modifications since its inception, and has greatly improved the quality of life of many children and adolescence with neurogenic bladders (Veeratterapillay et al. 2013; Farrugia and Malone 2010). It means that the child can self catheterize the Mitrofanoff at regular intervals – at least four times daily, thus obviating the need for use of nappies or diapers while remaining dry.

Ureteral substitution is rarely needed in children (Martin 1981; Estevao-Costa 1999; Dagash et al. 2008). Noted indications for its use include traumatic ureteric avulsion, congenital ureteric stenosis and ureteric obstruction following previous pyeloplasty for pelviureteric junction obstruction (Estevao-Costa 1999; Dagash et al. 2008). The tip of the appendix is usually discarded, the lumen irrigated and the mesoappendix widely dissected. The ends of the ureter and appendix are then spatulated and a single layer, end-to-end anastomosis created using polyglycolic sutures of an appropriate size. The success rate associated with this procedure is generally high (Martin 1981; Estevao-Costa 1999; Dagash et al. 2008).

Use of the appendix graft as a biliary conduit is the most recent, growing surgical use of the appendix. Though its use for patients with biliary atresia has been questioned and suggested only as a salvage technique, (Delarue et al. 2000) its use in the management of children with choledochal cysts- which has a variable incidence of 1 in100,000 to 150,000 in western countries to a much higher incidence of 1 in 1000 in Japan, (Gonzales and Lee 2012) appears to be gaining acceptance (Sarin et al. 2007; Shah and Shah 2005). At laparotomy the choledochal cyst is excised and the caecum and ascending colon fully mobilized so that the appendix is brought out of the right iliac fossa and into the right upper quadrant. The appendix is then divided at its base while carefully preserving the appendicular artery. After patency of the appendix is confirmed the wider caecal end of the appendix is anastomosed to the common hepatic duct in an end- to end manner. The opposite end of the appendix is then anastomosed to the posterior aspect of the second part of the duodenum in an end-to-side fashion. Advantages of its use in these children include utilization of a simpler technique which is less time-consuming, fewer suture lines, decreased episodes of postoperative cholangitis and the ability to allow postoperative evaluation using ERCP (endoscopic retrograde cholangiopancreatography) which is not possible with other bilioenteric procedures (Valla 1988; Shah and Shah 2005; Delarue et al. 2000).

It is thus quite fortunate that an absent appendix is a rare phenomenon given the increasing surgical uses of this vestigial structure.

## Consent

Written informed consent was obtained from the patient’s guardian/parent/next of kin for the publication of this report and any accompanying images.
